# Analysis of the trends in burden of meningitis in China from 1990 to 2021, and projections until 2036

**DOI:** 10.3389/fpubh.2025.1603244

**Published:** 2025-07-30

**Authors:** Xi Xu, Kai Xu, Qiulin Wu

**Affiliations:** ^1^Department of Clinical Laboratory, Wenzhou People’s Hospital, Wenzhou, China; ^2^Department of Clinical Laboratory, The Second People’s Hospital of Lishui, Lishui, China

**Keywords:** meningitis, global burden of disease, incidence, prevalence, mortality, disability-adjusted life years

## Abstract

**Background:**

This study aimed to describe the temporal trends in age and sex burdens of meningitis in China from 1990 to 2021 and to compare them with the global burden of the disease.

**Methods:**

Using data from the Global Burden of Disease (GBD) 2021, this study analyzed the features of meningitis burden in China and globally, including incidence, prevalence, mortality, disability-adjusted life years (DALYs), age-standardized incidence rate (ASIR), age-standardized rates of mortality (ASMR), age-standardized rates of prevalence (ASPR), and age-standardized DALY rate (ASDR). The average annual percentage change (AAPC) and associated 95% confidence interval (95% CI) were computed using Joinpoint. A comprehensive comparative analysis of the differences in meningitis burden between China and the world was conducted from multiple dimensions, including age, sex, and periods. Using the BAPC and INLA software tools to perform a Bayesian Age-Period-Cohort (BAPC) analysis in R.

**Results:**

From 1990 to 2021, the ASIR fell from 66.565/100,000 to 31.649/100,000 globally, and from 30.833/100,000 to 5.791/100,000 in China. The ASPR decreased from 11,291,266/100,000 to 92.251/100,000 globally, and from 140.1/100,000 to 16.622/100,000 in China. The ASMR decreased from 7.416/100,000 to 2.947/100,000 globally, while decreased from 3.537/100,000 to 0.475/100,000 in China. The ASDR decreased from 551.176/100,000 to 208.565/100,000 globally, while decreased from 267.304/100,000 to 27.952/100,000 in China. The global AAPC of ASIR, ASPR, ASMR, and ASDR was −2.364, 2.679, −2.970%, and −3.131%, whereas in China it was −5.307, −6.665%, −6.311%, and −7.149, respectively. The effects of age and sex on the burden of meningitis were different. Men had higher incidence and mortality rates of meningitis than women. ASIR in males and females would decrease in the following years.

**Conclusion:**

There was a discernible decline in the incidence, prevalence, mortality, and DALYs associated with meningitis in China and worldwide between 1990 and 2021. Meningitis is more common and fatal in infants and early children, followed by the older adult. Men are more likely than women to die from meningitis and to contract it. It is anticipated that the disease burden of meningitis will continue to decline over the ensuing 12 years. Meningitis continues to be a significant public health concern in China due to the country’s huge and aging population.

## Introduction

Meningitis, an inflammatory central nervous system disorder with diverse etiologies including infectious (bacterial, viral, fungal) and non-infectious (autoimmune, iatrogenic) origins, remains a critical global health priority due to its severe clinical sequelae and socioeconomic ramifications (1–3). While viral meningitis demonstrates higher incidence with lower mortality, bacterial variants exhibit disproportionate lethality, with survivors frequently developing persistent neurological impairments ranging from sensorineural deficits to neurocognitive dysfunction (4, 5). The condition imposes multilevel socioeconomic burdens through acute care costs, chronic disability management, and productivity losses, prompting WHO’s 2030 Global Roadmap emphasizing epidemic containment via vaccination, mortality reduction through pathogen-specific interventions, and post-infectious rehabilitation protocols (6, 7). This multinational initiative emphasizes comprehensive surveillance systems, equitable vaccine distribution, and standardized neurorehabilitation strategies to address the multidimensional challenges posed by meningitis.

Meningitis persists as a critical global health challenge despite prophylactic and therapeutic advancements. Its burden arises from a multidimensional risk architecture encompassing environmental particulates (PM2.5/indoor pollutants), perinatal susceptibilities (maternal undernutrition, preterm delivery), and socioeconomic gradients operationalized through Socio-demographic Index (SDI) stratification (8, 9). Geospatial stratification reveals inverse SDI correlations, with hyperendemic transmission clusters concentrated in resource-limited settings, notably hyperendemic zones within Sub-Saharan Africa’s meningitis belt (10–13). Although diagnostic advancements and minimally invasive therapies have modified global epidemiological patterns, persistent surveillance gaps and heterogeneous healthcare access necessitate localized burden assessments.

China experienced five major meningitis pandemics in the pre-vaccine era, recurring every 8–10 years and lasting 3–4 years each (14, 15). Historically dominated by *Neisseria meningitidis* serogroup A (69.2% of isolates, 1956–2002), a pivotal epidemiological shift occurred with the emergence of the hypervirulent serogroup C ST4821 clone during Anhui’s 2003–2004 outbreak, which was associated with higher mortality (16). By 2005–2012, serogroup C (47.1%) had surpassed serogroup A (35.3%) among confirmed cases, with most serogroup C and some serogroup B strains belonging to the ST4821 complex (17). Vaccine interventions—specifically the introduction of the group A polysaccharide vaccine (MPV-A) in 1984 and its subsequent inclusion in the National Immunization Program (NIP) alongside the group A and C polysaccharide vaccine (MPV-AC) in 2007—significantly reduced meningococcal incidence and prevented large-scale outbreaks (18). Newborns and children under five remain the highest-risk groups (19). Despite low overall incidence, China’s vast population results in an absolute number of meningitis deaths comparable to high-burden nations in the African meningitis belt (e.g., Nigeria, Ethiopia), placing its absolute burden among the world’s highest (20). Recent surveillance indicates critical challenges from other pathogens: *Streptococcus pneumoniae* (responsible for 46.9% of pediatric bacterial meningitis cases during 2014–2016 and dominant in northern China) and *Staphylococcus aureus* (prevalent in southern China) have become predominant bacterial causes (21), while enteroviruses constitute >31.5% of viral meningitis cases in southern provinces like Guangxi (22).

Current epidemiological investigations utilizing GBD datasets have primarily emphasized macrolevel epidemiological evaluations, employing multidimensional frameworks to quantify secular trends in infectious meningitis burden through integrated analysis of incidence, mortality, and DALYs (23, 24). While these investigations elucidate global trajectory projections, they exhibit limited granularity in addressing region-specific epidemiological heterogeneities. China’s demographic magnitude (18.6% global population) and distinct immunization landscape necessitate dedicated burden characterization. This study addresses this gap through a dual analytical approach: (1) Comparative analysis of Chinese versus global meningitis burden (1990–2021) to systematically model temporal inflection points and age-sex stratified epidemiological transitions; (2) BAPC projections extending to 2036 for precision forecasting. The aim is to inform meningitis prevention prioritization, optimize vaccine deployment strategies, and guide equitable health resource distribution in alignment with China’s public health objectives.

## Methods

### Overview and data gathering

This analysis utilized the GBD 2021 dataset, which systematically quantifies health loss across 204 nations and territories categorized into 21 geographical regions, encompassing 369 diseases/injuries and 88 risk factors (25). Meningitis burden estimates (1990–2021) were derived from GBD’s standardized epidemiological metrics - including incidence, prevalence, mortality, and DALYs with corresponding age-standardized rates (ASRs) - accessed through the Global Health Data Exchange (GHDx) platform (https://vizhub.healthdata.org/gbd-results/). The GBD 2021 study’s methodological framework for multisource data integration, encompassing vital registration systems, surveillance reports, and scientific literature, has been rigorously documented (26, 27), ensuring comparability in temporal and spatial disease burden assessments.

### Data analysis

This study extracted meningitis burden metrics from the GBD database, including crude incidence rate (CIR), crude prevalence rate (CPR), crude mortality rate (CMR), and crude DALY rate (CDR) for each age group and corresponding age-standardized rates (ASIR, ASPR, ASMR, ASDR) for China and global populations. Temporal trend analysis was conducted through Joinpoint Regression (Version 4.9.1.0, National Cancer Institute) to calculate AAPC with 95% confidence intervals. For predictive modeling, BAPC analysis was implemented via R-based integrated nested INLA to project sex-specific ASIR trends from 2022 to 2036 (28).

## Result

### Incidence of meningitis in China and worldwide

China demonstrated a 79.41% cumulative incidence reduction in meningitis cases, declining from 333,405 (95% CI:262,416–408,765) in 1990 to 68,619 (95% CI:57,310–82,623) in 2021, paralleled by a global 40.55% decrease (3,809,554–2,264,858 cases). ASIR exhibited accelerated declines in China (30.833 to 5.791/100,000) compared to global trends (66.565 to 31.649/100,000). In the meantime, the AAPC of the incidence rate in China declined to 5.307% (95% CI: −5.735 to −4.877), exceeding the global AAPC of 2.364% (95% CI: −2.449 to −2.278) over the study period (Table 1).

**Table 1:** All-age cases and age-standardized incidence, prevalence, mortality, and DALYs rates and corresponding AAPC of meningitis in China and globally in 1990 and 2021.

Location	Measure	1990		2021		1990–2021 AAPC
		All-age cases	Age-standardized rates per 100,000 people	All-age cases	Age-standardized rates per 100,000 people	
		*n* (95%CI)	*n* (95%CI)	*n* (95%CI)	*n* (95%CI)	*n* (95%CI)
China	Incidence	333,405 (262,416–408,765)	30.833 (24.846–37.426)	68,619 (57,310–82,623)	5.791 (4.789–7.111)	−5.307 (−5.735 to −4.877)
	Prevalence	1,646,604 (1,306,354–20,86,178)	140.1 (110.621–177.692)	256,233 (200,559–337,638)	16.622 (13.095–21.777)	−6.665 (−6.786 to −6.543)
	Deaths	37,283 (30,283–45,375)	3.537 (2.904–4.273)	6,176 (5,283–7,105)	0.475 (0.414–0.537)	−6.311 (−6.512 to −6.109)
	DALYs	2,991,866 (2,360,958–3,648,770)	267.304 (210.394–326.381)	285,432 (247,409–320,679)	27.952 (23.734–32.228)	−7.149 (−7.480 to −6.816)
Global	Incidence	3,809,554 (3,213,533–4,481,318)	66.565 (56.928–76.95)	2,264,858 (1,991,578–2,568,203)	31.649 (27.784–35.965)	−2.364 (−2.449 to −2.278)
	Prevalence	11,291,266 (9,145,095–14,249,891)	214.103 (172.984–270.788)	7,273,647 (5,928,780–9,065,624)	92.251 (75.193–114.792)	−2.679 (−2.799 to −2.558)
	Deaths	420,141 (370,817–482,318)	7.416 (6.592–8.43)	213,962 (175,653–265,664)	2.947 (2.383–3.705)	−2.970 (−3.062 to −2.878)
	DALYs	33,007,168 (28,883,871–38,409,475)	551.176 (484.269–638.803)	14,511,046 (11,401,702–18,634,697)	208.565 (161.845–270.172)	−3.131 (−3.229 to −3.033)

### Prevalence of meningitis in China and worldwide

China experienced an 84.44% cumulative prevalence reduction in meningitis cases, decreasing from 1,646,604 (95% CI:1,306,354–2,086,178) in 1990 to 256,233 (95% CI:200,559–337,638) in 2021, while globally, prevalence declined 35.58% (11,291,266–7,273,647 cases). ASPR demonstrated accelerated reductions in China (140.100 to 16.622/100,000) compared to global trends (214.103 to 92.251/100,000) during this period. China demonstrated a more rapid reduction in meningitis prevalence, exhibiting AAPC of −6.665% (95% CI: −6.786 to −6.543) from 1990 to 2021, compared to the global AAPC of −2.679% (95% CI: −2.799 to −2.558) during the same period (Table 1).

### Mortality of meningitis in China and worldwide

China achieved a 49.07% mortality reduction in meningitis-related deaths between 1990 and 2021, with the ASMR decreasing from 3.537 (95% CI: 2.904–4.273) to 0.475 (95% CI: 0.414–0.537) per 100,000 people. Globally, ASMR declined from 7.416 (95% CI:6.592–8.430) to 2.947 (95% CI: 2.383–3.705) per 100,000 people during this period. In 2021, meningitis caused 213,962 global deaths (95% CI:175,653–265,664), representing a 15.6% reduction from 1990. China achieved a 2.13-fold faster-annualized mortality reduction in meningitis compared to global trends, with an AAPC of −6.311% (95% CI: −6.512 to −6.109) versus −2.970% (95% UI: −3.062 to −2.878) between 1990 and 2021 (Table 1).

### DALYs of meningitis in China and worldwide

Global meningitis DALYs decreased by 56.04% (33,007,168–14,511,046) between 1990 and 2021, while China achieved an 84.44% reduction. ASDR declined from 551.176 (95% CI:484.269–638.803) to 208.565 (95% CI:161.845–270.172) per 100,000 people globally, versus China’s sharper reduction from 267.304 (95% CI: 210.394–326.381) to 27.952 (95% CI: 23.734–32.228) per 100,000 people. Furthermore, the AAPC of DALYs in China decreased by −7.149% (95% CI: −7.480 to −6.816), which was 2.28 times the global decline. The above data indicate significantly superior progress across incidence, prevalence, mortality, and DALY metrics in China compared to global averages (Table 1).

### Joinpoint regression nalysis of the burden of meningitis in China

**Figure 1:**
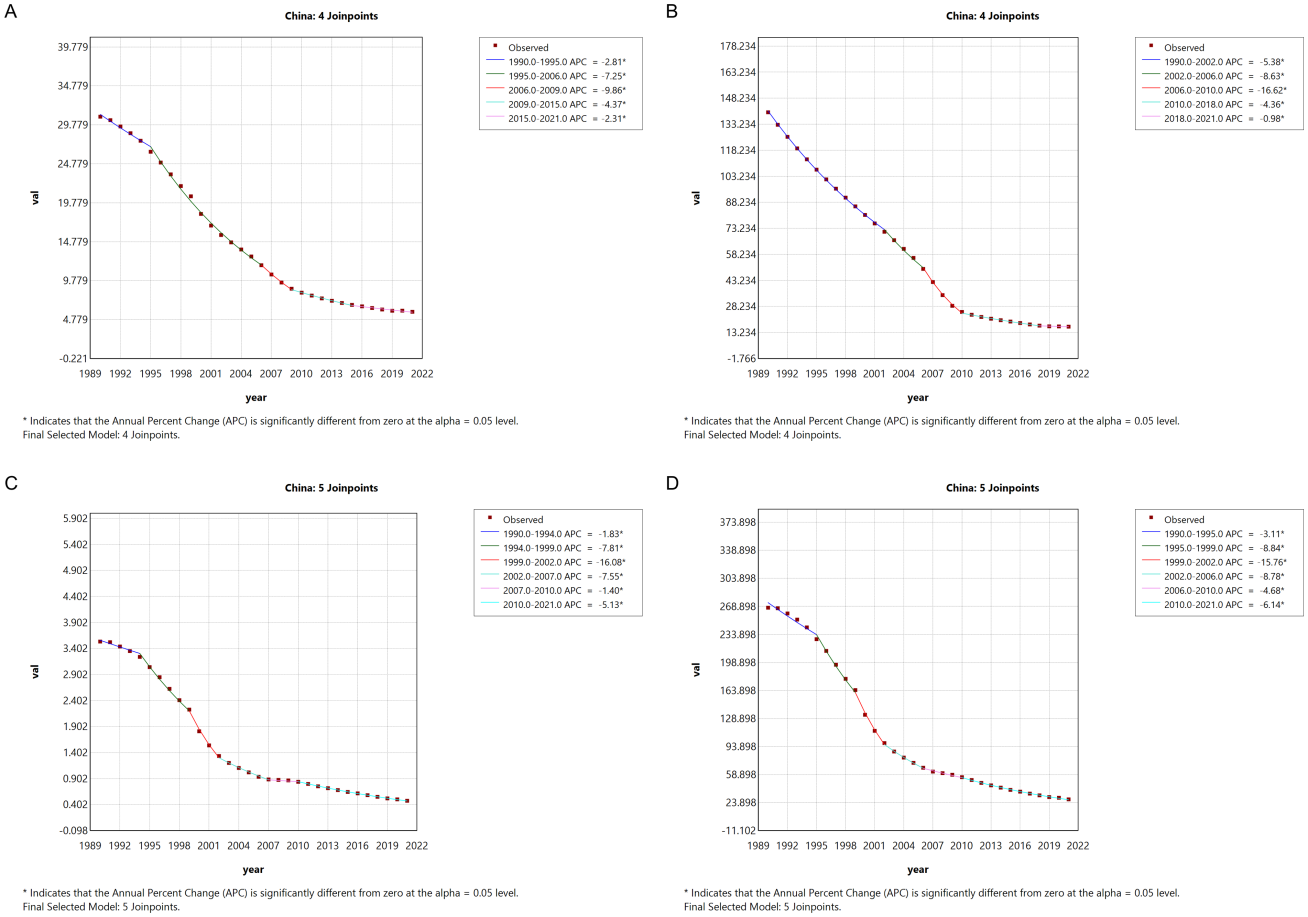
The APC of ASIR, ASPR, ASMR, and ASDR of meningitis in China from 1990 to 2021 (* means *p* < 0.05 and significant results). **(A)** ASIR; **(B)** ASPR; **(C)** ASMR; **(D)** ASDR.

The Joinpoint regression analysis of meningitis epidemiology in China from 1990 to 2021 reveals significant downward trends in ASIR, ASPR, ASMR, and DALYs. The APC for meningitis ASIR in China exhibited a progressive acceleration (ASIR: 1990–1995 APC = −2.81; 1995–2006 APC = −7.25; 2006–2009 APC = −9.86). The subsequent deceleration in reduction velocity post-2009 did not reverse the downward trajectory, with sustained negative growth persisting annually. The APC for ASPR in China exhibited parallel decline patterns, with accelerated reduction phases: −5.38 (1990–2002), −8.63 (2002–2006), and −16.62 (2006–2010). Mortality indicators showed sustained improvements, with ASMR decreasing at APCs of −7.81 (1994–1999), −16.08 (1999–2002), and −7.55 (2002–2007). Similarly, ASDR declined at −8.84 (1995–1999), −15.76 (1999–2002), and −8.78 (2002–2006) (Figure 1).

**Figure 2:**
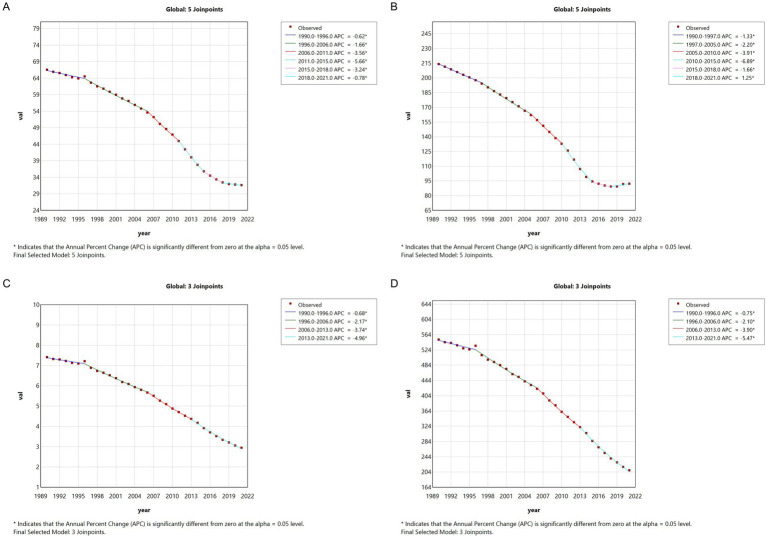
The APC of ASIR, ASPR, ASMR, and ASDR of meningitis in global from 1990 to 2021 (* means *p* < 0.05 and significant results). **(A)** ASIR; **(B)** ASPR; **(C)** ASMR; **(D)** ASDR.

Global meningitis metrics displayed comparable downward trajectories, though with distinct temporal patterns. ASPR decreased from 2005 to 2015 (APC = −3.91, 2005–2010; APC = −6.89, 2010–2015), followed by a marginal resurgence (APC = 1.25, 2018–2021). The consistent negative APC trends across all measured indices from 1990 to 2021 confirm significant epidemiological transition patterns in both Chinese and global contexts (Figure 2).

### Trends in the burden of meningitis disease in China

**Figure 3:**
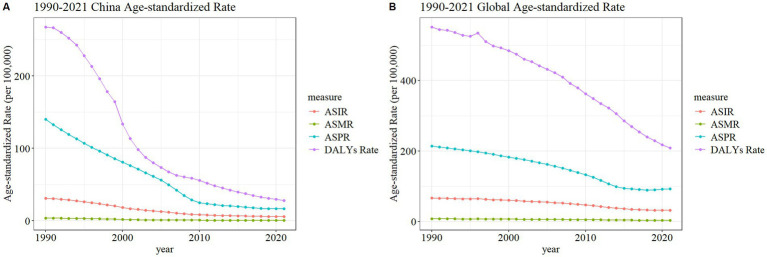
Trend comparison of ASIR, ASPR, ASMR, and ASDR of meningitis in China **(A)** and worldwide **(B)** from 1990 to 2021.

The ASDR for meningitis demonstrated sustained declines in both China and global contexts from 1990 to 2021, with China exhibiting a substantially greater reduction magnitude. Concurrently, the ASPR in China underwent marked decreases from 1990 to 2010, followed by trend stabilization post-2010. In contrast, global meningococcal ASPR maintained a plateau during 1990–2010, with subsequent moderate reductions. ASIR and ASMR showed limited yet consistent decreases in both regions throughout the study period (Figure 3). These different trajectories highlight the differences in the implementation effects of preventive intervention and disease surveillance systems between China and the global average.

### Burden of meningitis in different age groups in China in 1990 and 2021

**Figure 4:**
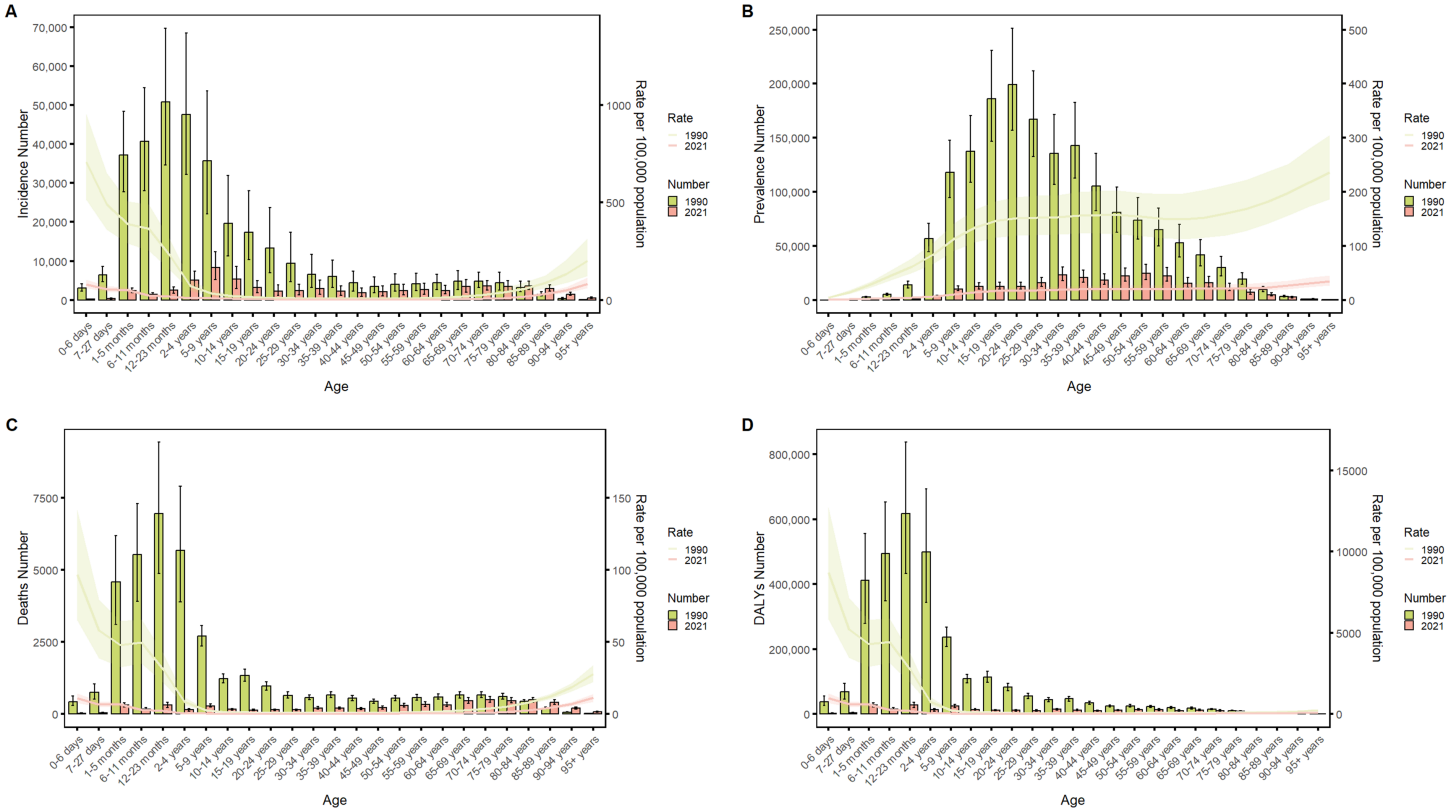
Comparison of China’s incidence, prevalence, mortality, and DALYs counts with their crude rates, by age group from 1990 to 2021. Bar plots show counts; lines show crude rates. **(A)** Incident cases and CIR; **(B)** Prevalent cases and CPR; **(C)** Death cases and CMR; **(D)** DALYs counts and CDR.

**Figure 5:**
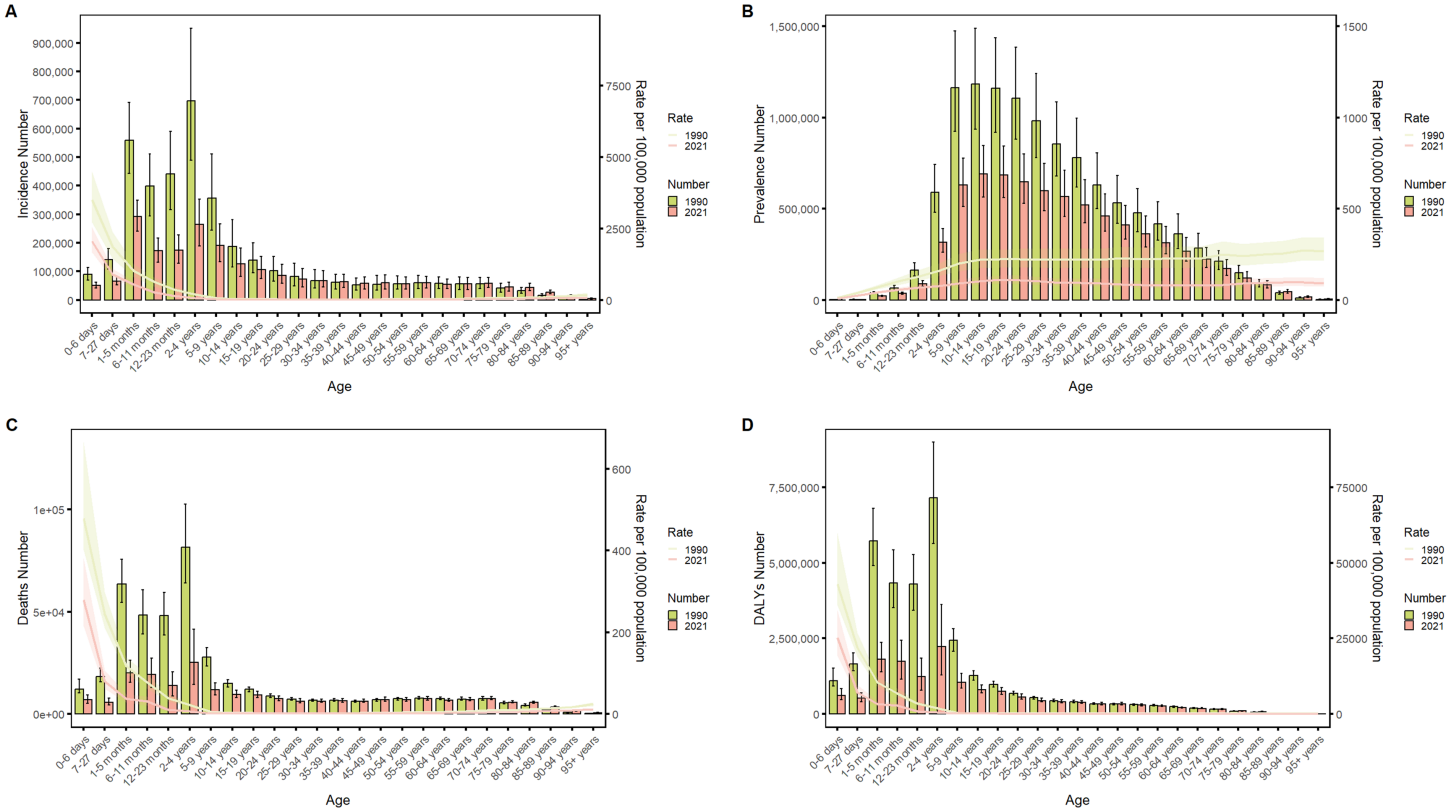
Comparison of the global incidence, prevalence, mortality, and DALYs counts with their crude rates, by age group from 1990–2021. Bar plots show counts; lines show crude rates. **(A)** Incident cases and CIR; **(B)** Prevalent cases and CPR; **(C)** Death cases and CMR; **(D)** DALYs counts and CDR.

Figure 4 delineates the age-specific epidemiological evolution of meningitis in China from 1990 to 2021, demonstrating dynamic shifts across incidence, prevalence, mortality, DALYs, and the corresponding crude rates. According to the incidence rate data, the predominant case burden is concentrated in the 1-month to 24-year cohort, particularly within the 1-month to 9-year subgroup. Temporal analysis revealed distinct phase transitions: the highest incidence peak shifted from 12–23-month infants (1990) to 5–9-year children (2021), while prevalence peaks migrated from 20–24-year young adults (1990) to 50–54-year middle-aged populations (2021). Mortality patterns exhibited a U-shaped curve, with the highest death peak transitioning from 12–23-month infants (1990) to 70–74-year elders (2021), while the highest mortality rates persisted in neonates (0–6 days, 1990) and nonagenarians (≥95 years, 2021). DALYs remained concentrated in 12–to 23-month infants across both epochs. The global trend parallels that of China but exhibits a less pronounced decline in magnitude (Figure 5). These data collectively illustrate an effective implementation of meningitis control strategies (vaccination, early diagnosis), while highlighting residual vulnerabilities in pediatric populations and emerging geriatric risks.

### Sex disparities in the burden of meningitis in different age groups in China

**Figure 6:**
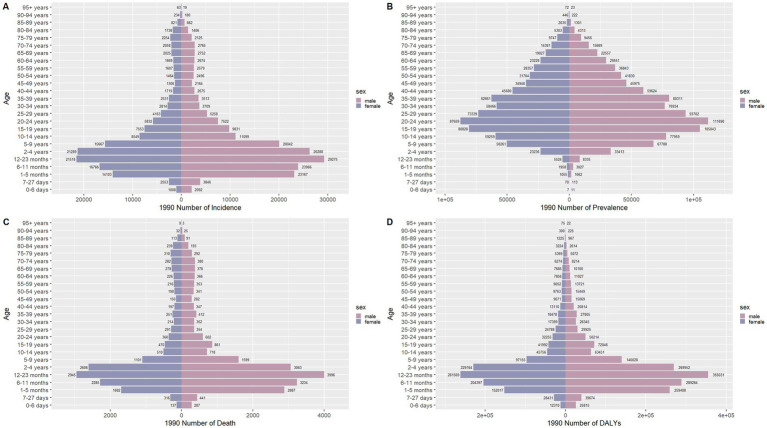
Comparison of the number of incidence, prevalence, mortality, and DALYs of meningitis in males and females of different age groups in China in 1990. **(A)** Incidence; **(B)** Prevalence; **(C)** Mortality; **(D)** DALYs.

**Figure 7:**
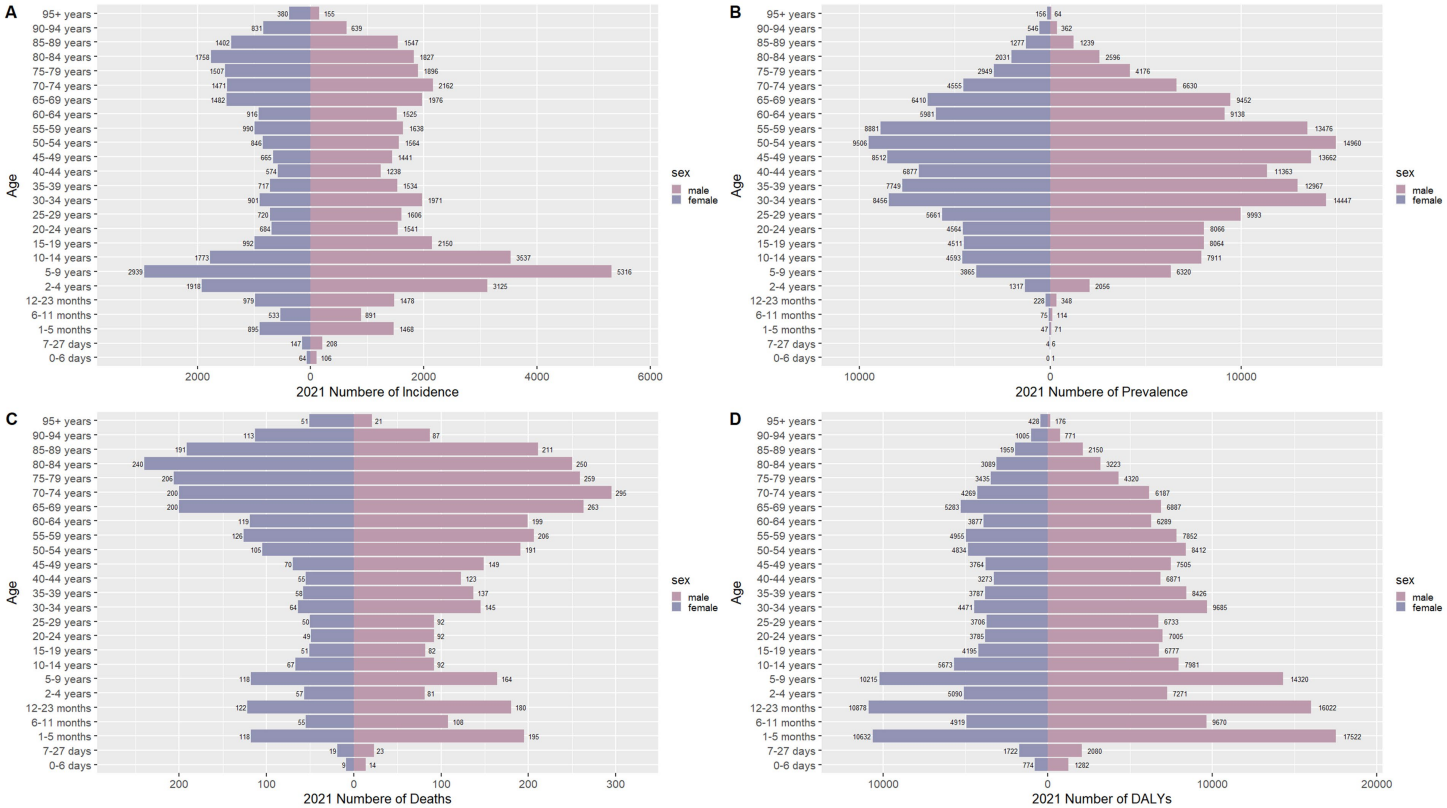
Comparison of the number of incidence, prevalence, mortality, and DALYs of meningitis in males and females of different age groups in China in 2021. **(A)** Incidence; **(B)** Prevalence; **(C)** Mortality; **(D)** DALYs.

**Figure 8:**
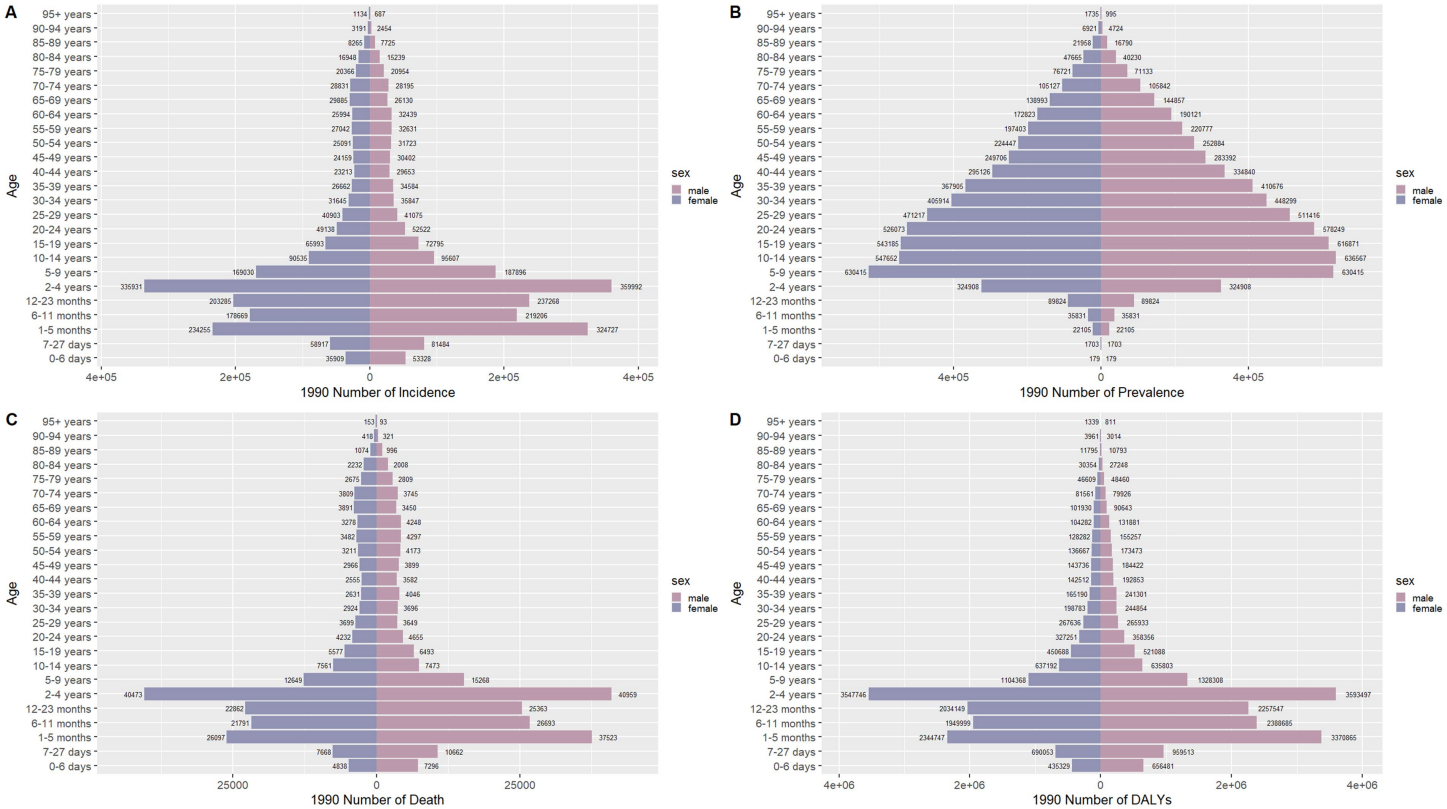
Comparison of the number of incidence, prevalence, mortality, and DALYs of meningitis in males and females of different age groups globally in 1990. **(A)** Incidence; **(B)** Prevalence; **(C)** Mortality; **(D)** DALYs.

**Figure 9:**
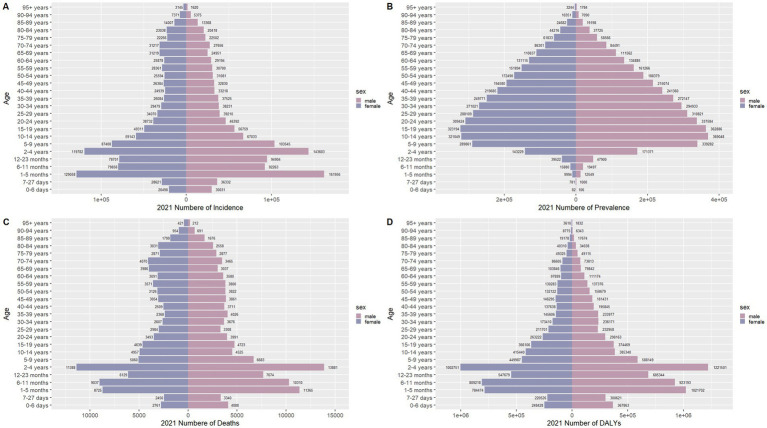
Comparison of the number of incidence, prevalence, mortality, and DALYs of meningitis in males and females of different age groups globally in 2021. **(A)** Incidence; **(B)** Prevalence; **(C)** Mortality; **(D)** DALYs.

**Figure 10:**
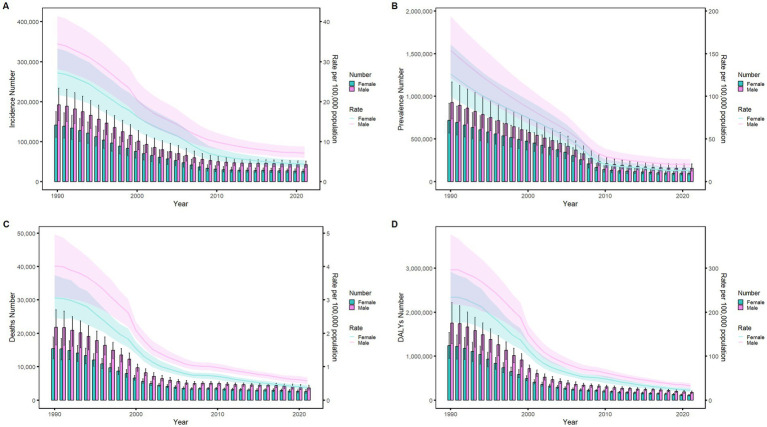
Comparison of age-standardized incidence, prevalence, death, and DALYs among Chinese men and women from 1990 to 2021. **(A)** Instances of incidents and ASIR; **(B)** Instances of prevalence and ASPR; **(C)** cases of deaths and ASMR; **(D)** counts of DALYs and ASDR. Bar charts show counts, and lines show rates normalized for age.

**Figure 11:**
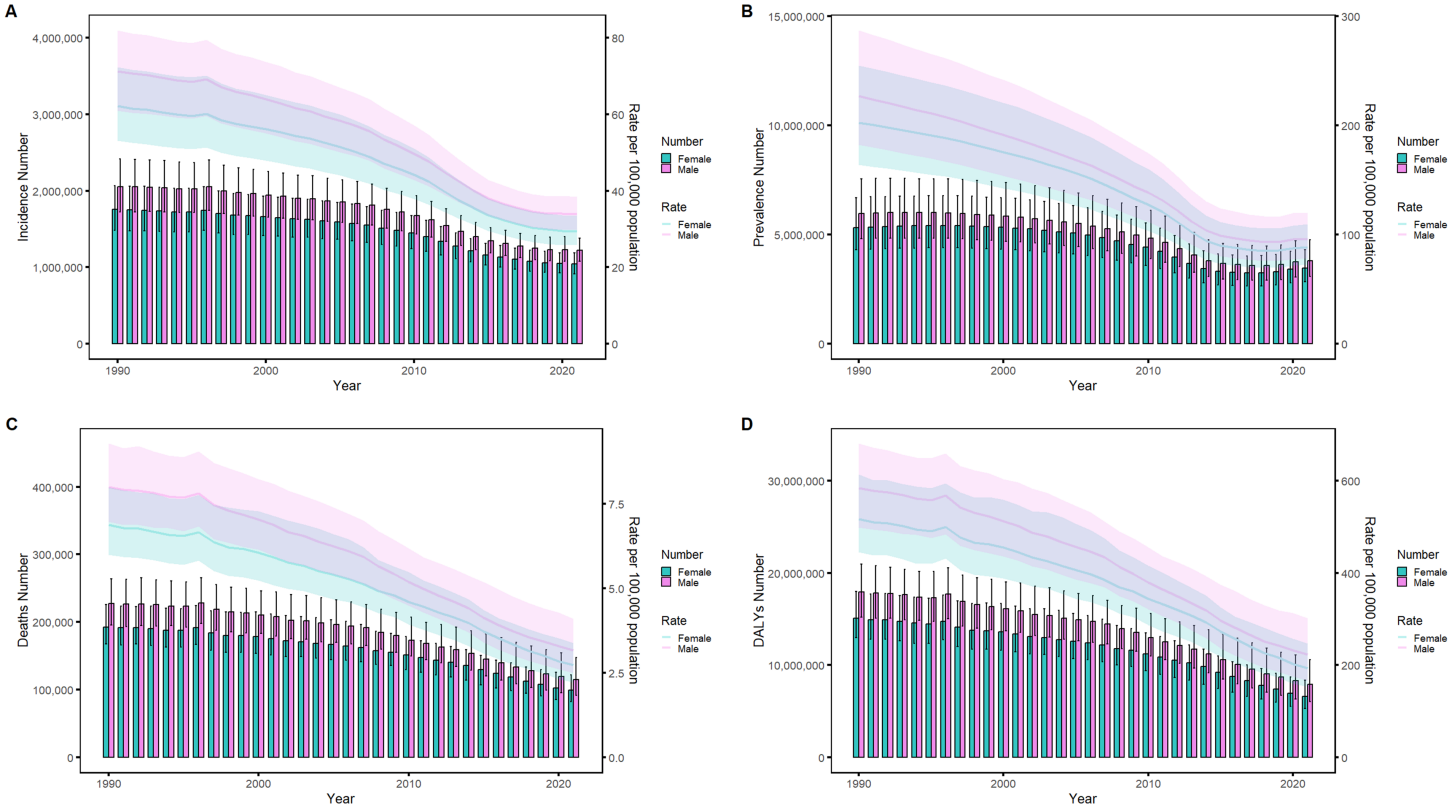
Comparison of age-standardized incidence, prevalence, death, and DALYs among men and women globally from 1990 to 2021. **(A)** Instances of incidents and ASIR; **(B)** Instances of prevalence and ASPR; **(C)** cases of deaths and ASMR; **(D)** counts of DALYs and ASDR. Bar charts show counts, and lines show rates normalized for age.

Figures 6–9 delineate sex- and age-stratified meningitis epidemiology in China and globally (1990 vs. 2021). In 1990, both sexes exhibited peak incidence at 12–23 months, followed by age-dependent decline until late-life resurgence (post-49 years), with male predominance across all sub-75 age groups. Epidemiological shifts by 2021 relocated the highest incidence burden to the 5–9-year demographic, with progressive geriatric increases beyond age 64 and sustained male preponderance in populations under 90 years. Prevalence patterns showed parallel sex-specific dynamics, peaking in young adults (20–24 years) in 1990 versus middle-aged populations (50–54 years) in 2021. Males had more cases than females in 1990 across all age categories under 74. By 2021, it had become the majority in the age group under 84 years old. Mortality demonstrated bimodal distribution: infantile peaks (12–23 months) in 1990 transitioned to geriatric maxima (Male:70–74 years, Female:80–84 years) in 2021. DALYs remained concentrated in infants throughout, though 2021 data showed sex-divergent peaks (Male:1–5 months, Female:12–23 months). ASIR, ASPR, ASMR and ASDR revealed synchronized declines with narrowing sex gaps in China (Figure 10), contrasting with globally persistent sex disparities and attenuated reductions (Figure 11). The above results indicate that age and sex have different effects on the burden of meningitis and also reflect differential implementation efficacy of prevention strategies between China and global populations.

### The projected burden of meningitis, 2021–2036

**Figure 12:**
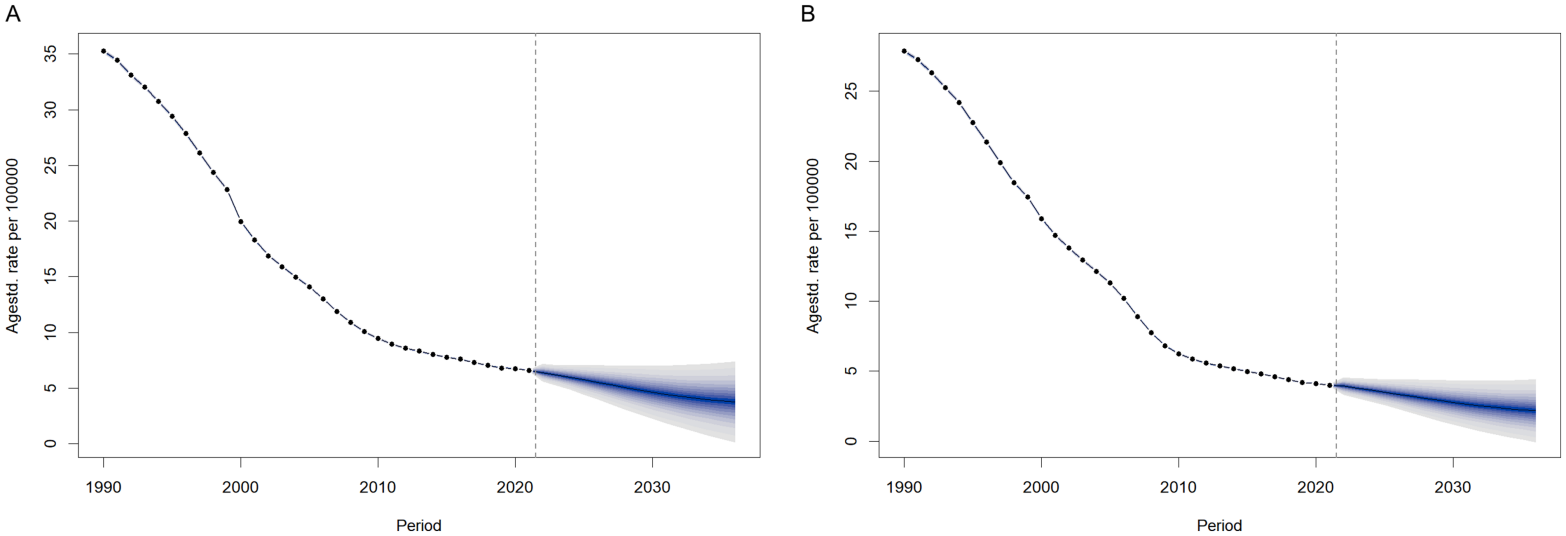
Trends of ASIR from 2021 to 2036 in males **(A)** and females **(B)** predicted by BAPC models.

We predicted the ASIR by sex from 2021 to 2036 using BAPC models in order to understand the trends of ASIR of meningitis beyond 2021. The findings are shown in Figure 12. After 2021, the ASIR for females would drop yearly from 4.00 per 100,000 (with a 95% CI of 3.95 to 4.05 per 100,000) in 2021 to 2.18 per 100,000 (with a 95% CI of −0.05 to 4.41 per 100,000) in 2036, as illustrated in Figure 12B. According to the forecast, the annual ASIR for males will drop from 6.60 per 100,000 (with a 95% CI of 6.53 to 6.60 per 100,000) in 2021 to 3.78 per 100,000 (with a 95% CI of 0.18 to 7.37 per 100,000) in 2036. The modeled trajectories suggest continued effectiveness of current prevention strategies and persistent sex-based disparities in disease burden. However, these projections assume stable age-period-cohort effects and exclude external factors like intervention updates or pathogen evolution. While the declining ASIR trend (with confidence intervals) supports current strategies, actual rates may vary if contextual factors diverge from historical patterns.

## Discussion

This study conducted a comprehensive analysis of meningitis burden in China and globally between 1990 and 2021 using GBD 2021 data. Age- and sex-stratified comparisons revealed consistent declines in ASIR, ASPR, ASMR, and ASDR, with China’s reduction rates surpassing global averages across all metrics. The incidence, prevalence, mortality, and DALYs of meningitis are all age-related, with children experiencing higher rates of both morbidity and mortality. The peaks of incidence rate, prevalence rate, and mortality rate have changed in China. In terms of sex composition, males were more susceptible to meningitis and had a higher risk of death after infection compared to females. Over the next 12 years, the disease burden of meningitis is expected to continue declining, according to projections made by the BAPC model. These findings underscore successful immunization scale-up while highlighting residual pediatric vulnerabilities and evolving prevention priorities.

Our observed decline in overall meningitis metrics align with the global downward trend reported in the recent GBD study (24), which can be attributed to concerted efforts over the past 30 years on meningitis disease control, such as enhancing healthcare resources, effective prevention and control measures, and international collaboration. Central to this progress has been the widespread implementation of conjugate vaccines against major causative agents, including *Haemophilus influenzae* type B (Hib), *Neisseria meningitidis* (serogroups A, C, W, Y), and *Streptococcus pneumoniae* (29–32).

Historically, Hib meningitis posed a significant threat, with 15–30% of infected children developing long - term sequelae such as hearing loss and neurological disabilities. However, since the 2000s, strategic interventions have transformed the landscape. The Global Alliance for Vaccines and Immunization (GAVI) played a pivotal role, providing critical support to resource - poor nations and significantly increasing the utilization of hepatitis B vaccines containing Hib (33, 34). In 2006, the WHO’s endorsement of worldwide Hib vaccination further propelled its adoption, leading to near - eradication in high - income countries and marked incidence reduction globally (35).

Parallel advancements in pneumococcal and meningococcal vaccination programs have also yielded impressive results. The pneumococcal conjugate vaccine (pCV7) was integrated into 26 national immunization programs, including one middle—income country, between 2000 and 2008; by December 2011, 77 countries either offered pCV7 universally or achieved over 50% coverage (36). In the African meningitis belt, the 2010 introduction of the MenA conjugate vaccine (MenAfriVac) triggered a dramatic 99% decline in confirmed MenA cases by 2015 (37–39). These vaccination efforts, complemented by antibiotic advancements reducing bacterial meningitis case - fatality rates, collectively drove the global epidemiological transition of meningitis burden (40). Notably, the timings of these immunization efforts align closely with trend analysis findings. The decline in the global ASPR from 2005 to 2015 in our finding corresponds directly to the rollout of vaccines, underscoring the significant impact of targeted vaccination programs on reducing the global meningitis burden.

Notably, the study reveals a resurgence in meningitis prevalence from 2018 to 2021, a period coinciding with global immunization disruptions caused by the COVID-19 pandemic. Widespread disruptions to healthcare services—including vaccination programs and disease surveillance—led to a significant decline in global childhood vaccination rates, leaving millions of children vulnerable to vaccine-preventable diseases (41). This disruption directly contributed to the observed increase in the global ASPR post-2018.

China’s structured immunization policies have been pivotal in reducing the meningitis burden. Since 1989, when meningococcal disease was classified as a notifiable Class B infection, a series of strategic interventions have been implemented. The universalization of the group A polysaccharide vaccine (MPV-A) in the 1980s laid the foundation for control efforts, particularly in rural areas prone to meningitis outbreaks. The inclusion of MPV-A in the National Immunization Program (NIP) in 2003 and MPV-AC in 2007 significantly accelerated progress (42). These measures led to a sharp decline in the ASIR from 2006 to 2009, demonstrating the effectiveness of expanded vaccine coverage. After 2009, the rate of decline in ASIR slowed, likely due to factors such as vaccination saturation, the emergence of non-vaccine serogroups (e.g., group C), or regional disparities in vaccine implementation.

Meningitis is most common in newborns and early children, then in the older adult, with a peak incidence that happens between 1 month and 9 years of age. This result might be explained by the fact that children and the older adult have weakened immune systems and less resistance. Meningitis is ranked sixth among the many diseases affecting disability-adjusted life years in children under the age of ten and has a significant impact on children’s healthy quality of life (43). As China’s population ages, the percentage of older adult individuals who are infected rises, while that of infants falls. The prevalence transition from young adults to older mirrors childhood vaccine recipients reaching adulthood with durable immunity. The results indicate that it is necessary to optimize the prevention strategies for specific ages, emphasizing the strengthening of the immunization program for the pediatric population and the monitoring system for the older adult population. The significantly higher incidence, prevalence, and mortality rates of meningitis observed among males compared to females likely arise from interconnected biological, socio-behavioral, and healthcare-access determinants. Biologically, androgen-mediated immune modulation may heighten male susceptibility to invasive bacterial pathogens. These inherent differences are further amplified by socio-behavioral risk gradients: males experience greater occupational pathogen exposure (e.g., mining, construction, or agricultural sectors). Moreover, females demonstrate heightened medical care-seeking propensity and stricter personal health standards, which collectively lower pathogen exposure opportunities and reduce treatment delays following infection.

As a High-middle SDI nation per GBD classification, China demonstrates exceptional meningitis control progress: its ASIR (5.791/100,000) significantly undercuts the group average (7.94/100,000) and rivals High-SDI regions (5.41/100,000) (24), reflecting successful vaccination policies, medical system reforms, and enhanced surveillance. This outperformance extends to mortality trends, where China achieved a 49.07% decline—surpassing global reductions—attributed to early diagnostics (e.g., rapid antigen testing), standardized antibiotic protocols, and a responsive national infectious disease reporting system. However, critical gaps persist: China’s ASMR (0.475/100,000) remains double that of High-SDI regions (0.24/100,000) (24), exposing deficiencies in acute care infrastructure. This divergence underscores a global pattern: while targeted interventions can transcend socioeconomic constraints, geodemographic and systemic barriers perpetuate burden disparities. In sub-Saharan Africa’s low-SDI regions (44), hyperendemic transmission constrains global progress, while limited access to acute treatment heightens neurological sequelae (45) and imposes catastrophic costs on households (46–49). Consequently, addressing persistent meningitis burden disparities worldwide requires geographically tailored strategies that integrate risk-based vaccination, strengthen acute care for vulnerable groups, and reduce financial barriers through health system reforms.

This study delivers robust, region-specific evidence that not only confirms the ongoing global decline in meningitis incidence but also reveals a distinct epidemiological trajectory for meningitis within China. The strength and novelty of these conclusions are underpinned by key methodological choices: First, the use of the globally standardized and high-quality GBD dataset provides a solid foundation, facilitating meaningful comparisons with international trends and ensuring our estimates are derived from a rigorous source. Second, the application of sophisticated BAPC models allowed us to disentangle the complex temporal dynamics driving meningitis burden over time, offering deeper insights than conventional analyses. Critically, this work addresses a significant gap by providing, to our knowledge, the first detailed longitudinal assessment of meningitis burden specifically focused on China, thereby offering novel evidence on its unique patterns and evolution in this context. However, it is also important to acknowledge the limitations of this study: First, diagnostic capacity constraints in resource-limited settings may underestimate true incidence through delayed/missed case ascertainment. Second, neonatal meningitis poses diagnostic complexities owing to non-specific neuroinflammatory manifestations that are frequently clinically indistinguishable from other neonatal comorbidities. Furthermore, temporal heterogeneity in surveillance methodologies and diagnostic criteria introduces potential secular bias.

## Conclusion

In summary, our analysis shows substantial global declines in meningitis burden from 1990 to 2021, with China’s integrated strategies of vaccination, early diagnosis, and rehabilitation offering scalable solutions for regions facing drug-resistant pathogens and climate-sensitive transmission. Looking ahead, these study results hold significant practical implications for public health policies and practices. As the meningitis burden is projected to decrease over the next 12 years, public health authorities can strategically optimize vaccination strategies by: (1) leveraging real-time pathogen surveillance data to dynamically adjust vaccine prioritization for emerging or prevalent serogroups; (2) allocating resources to promote combination vaccines that target multiple serogroups simultaneously; and (3) implementing adaptive vaccination schedules based on age-specific disease epidemiology—such as introducing booster doses for adolescents in areas with meningococcal outbreaks. Additionally, enhanced monitoring for high-risk groups like infants, children, and the older adult, through measures such as optimized pediatric vaccination schedules, supplementary drives in low-coverage areas, regular older adult health screenings, and provider training for early symptom recognition, combined with improved data collection in resource-limited settings, will enable accurate trend-tracking, intervention evaluation, and continuous refinement of public health approaches.

## Data Availability

Publicly available datasets were analyzed in this study. This data can be found here: https://vizhub.healthdata.org/gbd-results/.
